# Impact of physiological factors on longitudinal structural MRI measures of the brain

**DOI:** 10.1016/j.pscychresns.2022.111446

**Published:** 2022-04

**Authors:** Uzma Zahid, Emily P Hedges, Mihail Dimitrov, Robin M Murray, Gareth J Barker, Matthew J Kempton

**Affiliations:** a Department of Psychosis Studies, Institute of Psychiatry, Psychology & Neuroscience, King's College London, United Kingdom; b Department of Forensic & Neurodevelopmental Sciences, Institute of Psychiatry, Psychology & Neuroscience, King's College London, United Kingdom; c Department of Neuroimaging, Institute of Psychiatry, Psychology & Neuroscience, King's College London, United Kingdom

**Keywords:** Longitudinal, MRI, Hippocampus, Lateral ventricle, Hydration, Caffeine

## Abstract

Longitudinal MRI is used in clinical research studies to examine illness progression, neurodevelopment, and the effect of medical interventions. Such studies typically report changes in brain volume of less than 5%. However, there is a concern that these findings could be obscured or confounded by small changes in brain volume estimates caused by physiological factors such as, dehydration, blood pressure, caffeine levels, and circadian rhythm. In this study, MRI scans using the ADNI-III protocol were acquired from 20 participants (11 female) at two time points (mean interval = 20.3 days). Hydration, systolic and diastolic blood pressure, caffeine intake, and time of day were recorded at both visits. Images were processed using FreeSurfer. Three *a priori* hypothesised brain regions (hippocampus, lateral ventricles, and total brain) were selected, and an exploratory analysis was conducted on FreeSurfer's auto-segmented brain regions. There was no significant effect of the physiological factors on changes in the hypothesised brain regions. We provide estimates for the maximum percentage change in regional brain volumes that could be expected to occur from normal variation in each of the physiological measures. In this study, normal variations in physiological parameters did not have a detectable effect on longitudinal changes in brain volume.

## Highlights

• Clinical longitudinal MRI studies report changes in brain volume of less than 5%.
• Physiological factors such hydration may also cause small changes in brain volume.
• These physiological factors may obscure or even confound clinical changes.
• We tested the effect of 5 physiological factors on brain volume in healthy people.
• No effects of physiological factors were detected on longitudinal changes in volume_._

## 1 Introduction

Longitudinal structural MRI studies are increasingly used to examine neurodevelopment, illness progression and the effect of medical interventions. These studies may focus on neurological illnesses or mental health disorders, such as illness progression in Alzheimer's disease (Márquez and Yassa, 2019) and schizophrenia (Dietsche et al., 2017), as well as looking at healthy aging and neurodevelopment (Ecker et al., 2015; Resnick et al., 2003). Intervention studies have included measuring the impact of an aerobic exercise on hippocampal volume in patients with Alzheimer's disease (Frederiksen et al., 2018), mindfulness on gray matter density in patients with Parkinson's disease (Pickut et al., 2013), and a five-day intervention of TMS on regional gray matter volume in healthy volunteers (May et al., 2007). Although *cross-sectional* structural MRI studies of psychiatric disorders show reasonably large case-control effect sizes (5–10%) in volume (Bromis et al., 2018; Kempton et al., 2011b), within-subject brain morphometry studies show smaller regional brain volume change. For example, a longitudinal meta-analysis by Olabi et al. (2011) reported differences in whole brain gray matter volume reduction of approximately 0.5% per year between patients with schizophrenia and healthy controls. If there are common physiological variables that affect brain volume, these could obscure the effects of disease, development or interventions that are being measured. It is therefore important to measure and understand the effects of physiological variables on changes in brain volume so they can be controlled for in clinical studies. Accounting for physiological changes could significantly increase sensitivity of future longitudinal MRI studies and increase the reliability of serial measures using structural MRI.

Research into physiological factors influencing brain volume has shown effects of hydration (Kempton et al., 2009), circadian rhythm (Spira et al., 2016), blood pressure (Gonzalez et al., 2015), caffeine intake (Lin et al., 2019), and smoking (Van Haren et al., 2010). Variations in these physiological parameters could add random error to longitudinal MRI studies, or worse, if these parameters co-occur with an illness, such as increased dehydration in dementia (Bickel et al., 2018) or increased smoking rates in schizophrenia (De Leon and Diaz, 2005), they could act as a confound, resulting in spurious case-control differences.

The purpose of the Precision in Neuroimaging study was, therefore, to determine real-world variation of the above physiological parameters and examine their effect on changes in brain volume of healthy subjects. We have used time of day to index circadian rhythm, although we acknowledge that time of day may relate to other non-physiological variables, in this paper for simplicity we use the term ‘physiological’ to refer to all the variables under investigation. Although earlier studies have examined the effect of extreme interventions on brain volume, such as lack of fluid intake for 16 h (Duning et al., 2005), the aim of the current study was to examine the effect of the ranges of normal variation likely to be seen in a typical longitudinal MRI study. This study focused on young adults because our clinical research is on mental health disorders where the typical age of onset is in late adolescence and early adulthood (Kessler et al., 2007). For MRI acquisition, we chose the recently developed third generation of the Alzheimer's Disease Neuroimaging Initiative (ADNI) protocol ADNI-III (Gunter et al., 2017); while the ADNI sequence was developed for use in patients with Alzheimer's disease, it is being widely used in international studies of mental health disorders [e.g., PSYSCAN (Tognin et al., 2020), NAPLS (Cannon et al., 2014); EU-GEI (Pollak et al., 2020)] and is likely to be used in future longitudinal neuroimaging studies. The Precision in Neuroimaging study has previously reported on the impact of image acquisition and processing factors on the reliability of structural brain measurements (Hedges et al., 2022).

When examining the effects of physiological factors on brain volume, a potentially large number of brain regions could be examined, leading to false positives. Therefore, this paper focused on changes of three *a priori* hypothesised brain volumes. Total brain, and lateral ventricle volume were selected as there is strong evidence for an effect of specific physiological variables on these brain regions. For example, we have previously shown that dehydration is associated with an increase in lateral ventricle volume (Kempton et al., 2009) and Biller et al. (2015) reported reduced brain volume during dehydration (Biller et al., 2015). Hippocampal volume was additionally selected as the hippocampus has been a major focus of research in both psychiatric and neurological illnesses (Fotuhi et al., 2012), but few studies have examined the influence of physiological factors on hippocampal volume.

However, to benefit future research, the results from an exploratory analysis of the effects of the physiological variables on a large number of regions are available in a supplement to this paper. In addition, defaced neuroimaging data and physiological measures for a subset of participants who provided consent has been made publicly available for researchers to download.

## 2 Methods

### 2.1 Participants

Subjects were recruited from the Institute of Psychiatry, Psychology & Neuroscience (IoPPN), King's College London, and from the general public. Exclusion criteria were a history of either a diagnosis of a mental health condition for which they received treatment, a neurological disorder such as Parkinson's or Huntington's disease, or a significant medical disorder. Furthermore, contraindication to an MRI environment excluded participants from the study. The project received approval from the King's College London Ethics Committee. Participants provided written consent prior to participation.

### 2.2 Overall design

After screening, participants attended the MRI unit on two occasions approximately three weeks apart. At three weeks, we don't expect to see any age-related changes on brain volume, but this follow-up period is long enough to observe variation in physiological measures (Dieleman et al., 2017).

At baseline and follow-up visits, participants had an MRI scan, completed a questionnaire, had their blood pressure measured and provided a urine sample. Two consecutive MRI scans were acquired at baseline (A_1_, A_2_), and at follow-up (B_1_, B_2_). Within-session variability was examined using A_1_ and A_2_ and between-session variability was examined using A_1_ and B_1_. In this study participants were not repositioned in the scanner and B_2_ was not used.

### 2.3 Physiological measures

On the day of the scan, participants were asked to self-report their caffeine intake (cups of caffeinated drinks) and their tobacco use (number of cigarettes). Participants also provided a urine sample to determine how hydrated they were. Urine was analysed on the same day using a refractometer which measures urine specific gravity (USG) (Clinical Refractometer ATC Tri-Scale Serum Protein 0–12 g/100 ml Urine Specific Gravity SG 1.000–1.050 Refractive Index 1.333–1.360RI). Blood pressure was measured by placing the cuff on the upper left arm while participants were seated (Omron Basic M2 Blood Pressure Measuring Device for Upper Arm). As only one participant was a smoker, smoking was removed as a variable from the final analysis.

### 2.4 MRI data acquisition

Structural MRI data was acquired using a 3T GE MR750 Discovery scanner (GE Healthcare, Chicago, USA) at the centre for Neuroimaging Sciences, King's College London. An accelerated Sagittal IR-FSPGR ADNI-III sequence was utilised for structural imaging data acquisition (Repetition Time (TR) = 7.36 ms, Echo Time (TE) = 3.05 ms, Inversion Time = 400 ms, Flip Angle (FA) = 11°). 196 sagittal slices with 1 mm slice thickness were acquired resulting in voxel dimensions of 1 × 1 × 1 mm. The ADNI-III sequence was chosen as it has been specifically developed for multicentre studies where homogeneity of tissue contrast and resolution between scanners is essential in order to achieve high accuracy, reliability, comparability and interpretability (Jack et al., 2008).

### 2.5 MRI data processing and analysis

FreeSurfer longitudinal pipeline (version 6.0.0; Martinos Center for Biomedical imaging) was run on the structural ADNI-III data using the *recon-all* command (Reuter et al., 2012). The pipeline consisted of bias field correction, Talairach transformation, initial tissue intensity normalization, skullstripping, subcortical structure labeling, white matter segmentation, generation of surface models, inflation of surfaces, mapping of inflated surface to spherical space, cortical parcellation and cortical parcellation statistics. Cortical global regions were derived from the Desikan-Killiany Atlas.

The data were screened for the presence of soft failures, i.e., skull strip, white matter segmentation and intensity normalization errors, as well as topological defects and pial surface misplacement. The latter were assessed by manual visual inspection following the protocol of Iscan et al. (2015) as well as the official FreeSurfer Troubleshooting Wiki page (http://freesurfer.net/fswiki/FsTutorial/TroubleshootingData).

The 3 *a priori* hypothesised regions explored in this study were: 1) hippocampus, 2) lateral ventricles (including the temporal horn), and 3) total brain (excluding ventricles). Additional FreeSurfer subcortical and cortical regions were analysed in an exploratory analysis (see Supplementary Materials for the results). To reduce the number of comparisons, left and right hemisphere volumes of the same anatomical structures were combined to give a total volume.

### 2.6 Statistical analysis

Absolute mean percentage difference (MPD) for brain regions and physiological measures were determined using the formula below, where *A*_1_ is the volume/measure at baseline and *B*_1_ is the volume/measure at follow-up and *n* is the number of participants:

$$\frac{\sum \left|\frac{A_{1}-\;B_{1}}{B_{1}}\right|*100}{n}$$

Percentage changes were calculated to demonstrate the amount of variation between participants but were not included in the subsequent statistical analysis. We have previously published intra-class correlation coefficients (ICC) on the current dataset (Hedges et al., 2022), and we provide these values in the current study for the hippocampus, lateral ventricles, and total brain volume.

Linear mixed models for repeated measures were fitted for each outcome of brain volume to examine the effect of physiological measures on changes in brain volume. Analyses were conducted in Stata statistical software, Stata/IC 16.0 (StataCorp, 2017). For each outcome and physiological predictor, a random-intercept model was fitted where a random effect of participant was specified to account for the covariances of the repeated measurements of individuals. A random slope of time was additionally added but was not retained for any of the models as likelihood ratio tests indicated that the simpler random-intercepts models fitted the data significantly better. Models were fit by maximum likelihood estimation (MLE). The number of days between scanning sessions was incorporated as a covariate in each model to account for variation in the number of days between visits. For the hypothesised brain regions, the total number of planned comparisons was 15 (5 physiological parameters x 3 brain regions) and therefore the Benjamini-Hochberg procedure, with a 5% false discovery rate (FDR) was adopted. In addition, results from the mixed models were used to calculate the maximum percentage change in brain volumes that could be expected to occur from normal variation in each of the physiological measures. We make available these results in the supplement (supplementary materials Section 3).

### 2.7 Data availability statement

Defaced structural MRI data and physiological measures have been made publicly available for a subgroup of participants who gave their consent for this  https://sites.google.com/view/pinstudy. Additional data is available from the authors on reasonable request, and such request may need approval from the King's College London Ethics Committee.

## 3 Results

### 3.1 Participants

Twenty healthy participants (11 female and 9 male; age range = 20 – 30 yrs; mean age = 24.0 SD = 2.9) completed the baseline and follow-up visit. The time between baseline and follow-up visit ranged from 5 to 80 days with mean interval duration of 20.3 days, median interval of 13.5 days and a single outlier (with an assessment interval of 80 days). As only one participant was a smoker, smoking was removed as a variable from the analysis.

### 3.2 Variation in physiological measures between visits

The mean baseline and follow-up values, the change between baseline and follow-up, and the MPD of the physiological variables are shown in Table 1. While the absolute percentage change is highly dependent on the type of scale used, it does indicate the observed within-subject variation.

**Table 1:** Baseline and follow up mean (and standard deviation) values with the MPD of the physiological variables. Change is calculated by follow-up minus baseline value for each individual and the range is shown in the change column. For example, for systolic blood pressure, in the change column the greatest increase was 11 mmHg, and the greatest decrease was 13 mmHg.

	Baseline measure		Follow-up measure		Change (baseline to follow-up, range)	MPD
	Mean	SD	Mean	SD		
**Hydration (USG)**	1.02	0.01	1.02	0.01	−0.03 to 0.02	0.75
**Systolic (mmHg)**	114.95	14.30	115.70	15.43	−13.00 to 11.00	4.51
**Diastolic (mmHg)**	75.30	9.58	72.55	8.31	−18.00 to 11.00	6.39
**Caffeine (cups)**	0.70	0.66	0.88	0.97	−1.00 to 2.00	47.50
**Time of Day (24 hour)**	12.50	2.67	12.31	2.70	−4.50 to 9.50	19.03

### 3.3 Variation in brain volumes between visits

Mean baseline and follow up volumes, with the MPD of the three key brain regions, are shown in Table 2. We have included the MPD between two consecutive scans carried out in the same scanning session at baseline (A_1_-A_2_) as well as the MPD between scan 1 at baseline and scan 1 at follow up (A_1_-B_1_). Volumes for the three hypothesised regions within the baseline session showed changes of 0.57% – 1.07% while changes after 3 weeks were only slightly higher with values between 0.75% and 3.69%. Graphs illustrating mean brain volume of the hypothesised brain regions for baseline and follow-up visits are provided in the supplement (see supplementary materials Section 1)

**Table 2:** Baseline and follow up volumes with the MPD and ICC values of the three key brain regions. ICC values are from our reliability analysis on the same dataset published in Hedges et al. (2022).

	Baseline volume (A_1_, ml)		Baseline volume (A_2_, ml)		A_1_ – A_2_		Follow-up volume (B_1_, ml)		A_1_ – B_1_	
	Mean	SD	Mean	SD	MPD	ICC	Mean	SD	MPD	ICC
**Hippocampus**	8.35	0.86	8.34	0.90	1.07	0.986	8.36	0.90	1.14	0.984
**Lateral Ventricles**	10.09	4.90	10.12	4.90	0.92	0.989	10.15	4.96	3.69	0.984
**Total Brain Volume**	1167.66	118.21	1170.26	118.89	0.57	0.998	1170.77	117.69	0.75	0.996

### 3.4 Effect of physiological variables on changes in brain volume

The effects of the physiological variables on the hypothesised brain regions are shown in Table 3. We found no significant effect of any of the physiological variables on hippocampal volume, lateral ventricles, and total brain volume. Our exploratory analysis of brain regions revealed some findings that individually reached significance (see supplementary materials Section 2), but these did not pass the required threshold for multiple comparisons.

**Table 3:** The effect of the physiological measures on changes in the a priori hypothesises brain regions.

	*p*-value	Coefficient^a^	95% Confidence interval	
**Total Brain Volume (ml)**				
Hydration	0.535	123.57	−266.41	513.55
Systolic Blood Pressure	0.909	−0.07	−0.76	0.62
Diastolic Blood Pressure	0.450	0.26	−0.42	0.94
Caffeine	0.054	4.91	−0.08	9.91
Time of Day	0.610	0.39	−1.10	1.88
**Hippocampus (ml)**				
Hydration	0.369	−2.22	−7.05	2.62
Systolic Blood Pressure	0.530	0.00	−0.01	0.01
Diastolic Blood Pressure	0.580	0.00	−0.01	0.01
Caffeine	0.813	0.01	−0.06	0.08
Time of Day	0.201	−0.01	−0.03	0.01
**Lateral Ventricles (ml)**				
Hydration	0.380	7.08	−8.72	22.88
Systolic Blood Pressure	0.120	0.02	−0.01	0.05
Diastolic Blood Pressure	0.994	0.00	−0.03	0.03
Caffeine	0.085	0.18	−0.03	0.39
Time of Day	0.114	0.05	−0.01	0.10

a 1 unit increase in the physiological measure will result in a change in total brain volume by the corresponding beta coefficient.

### 3.5 Range of percentage change in hypothesised brain regions from physiological variables

Although findings in the present study indicate that physiological variables did not have a substantial effect on brain volume, we have estimated the maximum percentage change in brain volumes that could be expected to occur from normal variation of such physiological variables (see supplementary materials Section 3).

## 4 Discussion

Overall, we found no substantial effect of hydration, blood pressure, caffeine, or time of day on longitudinal structural MRI measures. Smoking was excluded from the analysis as there were not enough smokers in our sample. In our exploratory analysis, we found no evidence of impact of the physiological variables on changes in brain structure that passed correction for multiple comparisons.

In addition, although the study reports no significant effect of physiological variables, the data allow us to estimate the maximum effect such variables could have on changes in brain volumes of our sample. These estimates suggest that the maximum effect would be typically less than 1% for total brain and hippocampal volume, but larger for the lateral ventricles. The more conservative estimates of potential brain changes arising from variation in the physiological variables were approximately double.

### 4.1 Hydration

Previous longitudinal studies investigating the effect of dehydration have found that increased dehydration is associated with decreases in brain volume. In a cross‐over and repeated measures study, Kempton et al., (2011a) examined the effect of acute dehydration on brain volume in 10 healthy adolescents and found ventricular enlargement positively correlated with the degree of dehydration. Biller et al. (2015) investigated the effect of hydration on brain morphometry using a longitudinal design with 15 healthy volunteers who were asked not to drink or eat meals containing more than 0.5 L of fluid for 12 h. MR imaging demonstrated decreases of cortical thickness and volumes of the whole brain, cortex, white matter, and hypothalamus/thalamus during the dehydration protocol and subsequent hydration resulted in an increase in global brain volume. In contrast to these previous studies, the aim of the current study was to examine the effects of normal variation of hydration in healthy adults and we did not detect a significant effect on any of the brain volumes investigated.

### 4.2 Blood pressure

There is strong evidence that *chronic* hypertension is associated with total brain volume and hippocampal reduction (Beauchet et al., 2013). In contrast, our study investigated within-subject normal variation of blood pressure in healthy volunteers and its impact on brain volume longitudinally. Previous studies have recorded MRI and blood pressure measures, but to our knowledge no studies have recorded both longitudinally. For example, Gonzales et al. (2015) investigated longitudinal changes in cortical thinning associated with hypertension and found that regions in the frontal and temporal cortices exhibited higher rates of thinning over time in those with hypertension. Yano et al., (2018) reported that visit-to-visit blood pressure variability in young adults was associated with smaller hippocampal volumes at the age of 51. Therefore, while there is evidence that blood pressure has an impact on brain volume, the current study suggests that the normal variation of blood pressure in healthy adults has a negligible impact on longitudinal measures of brain volume over a short period.

### 4.3 Caffeine

While several studies have explored the effects of caffeine on brain activity, there is little evidence looking at its effect on brain volume changes. In a cross-sectional study, Perlacki et al. (2011) investigated the relationship between caffeine intake and brain morphology in a cohort of 45 healthy women aged 19–30. Results showed that high and low-level caffeine intake was associated with a larger hippocampus compared to moderate-level caffeine intake. In a longitudinal study, Lin et al. (2019) investigated whether long-term caffeine intake alters gray matter structures through changes in sleep homeostasis in 20 healthy participants. The results showed that total gray matter volume was lower in the caffeine condition compared to placebo. The current study did not detect an effect of normal variations in caffeine over a short period of time on the hypothesised brain regions.

### 4.4 Time of day

In terms of the effect of time of day of scan on changes in brain structure, MacLaren et al. (2014) found that one of three subjects who were scanned approximately 40 times across 31 days during different hours of the day, showed a significant increase in ventricular volume from 8am to 10pm. Conversely, Nakamura et al. (2015) in a longitudinal study retrospectively analysing 3 large MRI datasets of 1589 participants found there was a statistically significant effect of time of day, showing that the brain volume is greater in the morning. Trefler et al. (2016) found a significant reduction in apparent brain volume from morning to evening in 19 healthy adult volunteers. In our study the difference in time of day of scan was relatively minimal compared to other studies therefore this could be the reason why no association has been found. However, we did not systematically control the time of day participants were scanned to reflect a normal study and were not able to detect a significant association, suggesting the effect is minimal.

### 4.5 Strengths and limitations

A strength of the current study is that it was prospectively designed to investigate the effects of a number of physiological variables concurrently. We have specifically focused on the impact of day-to-day variability of a range of physiological variables to ensure that our findings are relevant for typical research studies of young adult participants. In order to overcome the confounding effects of age-related changes that occur in the brain, our study had a mean interval duration of 20.3 days between visits. Furthermore, our study enables researchers to estimate the upper limit of the effect of these variables on changes in brain morphometry which will be useful in designing future studies and determining if physiological measures need to be controlled, measured, or even ignored. The ADNI-III T1 sequence was used as the ADNI protocol is widely used and the third generation of the sequence is likely to be used in future international studies. The ADNI-III sequence has been specifically developed for multicentre study utilization where homogeneity of tissue contrast and resolution between scanners is essential in order to achieve high accuracy, reliability, comparability and interpretability (Jack et al., 2008). We have used the FreeSurfer pipeline which is one of the most used analysis techniques so that the results of this study can inform a wide range of research groups who use this software. Furthermore, we chose to use the FreeSurfer longitudinal pipeline as we were principally concerned with the effect of normal variation in physiological factors on longitudinal changes in brain volume. As such the results of this study will be less informative for cross-sectional designs where normal variation in physiological factors may be less of a concern. Finally, in addition to between session variability from baseline to follow-up, we have also provided values of within session variability.

In regard to limitations, the study may have been underpowered to detect small effects of physiological factors which could still be relevant for large studies. However, our sample size of twenty participants is comparable to longitudinal studies discussed earlier (Biller et al., 2015; Kempton et al., 2011a; Lin et al., 2019; Maclaren et al., 2014; Trefler et al., 2016), as well as reliability studies investigating MRI acquisition parameters. For example, Brown et al. (2020) investigated the test-retest reliability of the FreeSurfer automated hippocampal subfield segmentation procedure with 11 healthy participants. Similarly, Melzer et al. (2020) assessed the reliability and reproducibility of three imaging modalities, one of which was structural MRI, with 20 healthy volunteers. Furthermore, we have made our data freely available (https://sites.google.com/view/pinstudy) allowing future investigators to combine this data with other imaging datasets to increase power. A further limitation was that for caffeine intake we relied on self-report. Self-report measures are widely used and are common tools to collect data, however, it is possible that participants may recall information inaccurately. Also, we did not carry out a urine drug test on participants and future studies should aim to do so in order to ensure findings are not confounded by substance abuse. Our study used one model of scanner and while this was important to reduce scanner-related variance, it could limit the generalisability of findings. Another limitation of the current study is that we did not account for factors which might affect USG, for example, use of drugs such as isotretinoin or increased secretion of anti-diuretic hormone (ADH). Our sample included adults aged 20 – 30 so the findings can be applied to samples of this age range, but further research will be needed to confirm if these findings can be applied to adolescents and older age groups. Additionally, our results cannot be generalised to groups with observed hypertension or dehydration as our sample is of young healthy people who had minimum variation in physiological measures. However, the aim of the current study was to determine real-world variation of the physiological parameters and examine their effect on changes in brain volume of healthy subjects.

### 4.6 Implications

This study holds positive implications for longitudinal MRI studies as we were not able to detect an effect of normal variations in hydration, blood pressure, caffeine, or time of day on regional brain volume changes. We have attempted to show the maximum possible effect of these variables on changes of brain volume and have estimated that these are typically around 1% or smaller. In contrast Alzheimer's disease atrophy rates are typically larger, a case-control meta-analysis of longitudinal studies reported the mean annualized hippocampus atrophy rate of 4.66%, and 1.41% for controls (Barnes et al., 2009). However, studies in other disorders such as schizophrenia have reported smaller values such as a 0.5% per year difference between patients with schizophrenia and healthy controls in gray matter volume (Olabi et al. 2011). Thus, for disorders where the change is less than 1%, researchers may need to consider the impact of physiological variables. Using the supplementary data from this publication, future studies will be able to check the brain regions they are interested in and verify if there is a concern with specific physiological variables as well as the magnitude of its affect. The data provided in this publication will allow investigators to select measure and control problematic variables in an informed and targeted way.

## Declaration of Competing Interest

The authors whose names are listed immediately below certify that they have NO affiliations with or involvement in any organization or entity with any financial interest (such as honoraria; educational grants; participation in speakers’ bureaus; membership, employment, consultancies, stock ownership, or other equity interest; and expert testimony or patent-licensing arrangements), or non-financial interest (such as personal or professional relationships, affiliations, knowledge or beliefs) in the subject matter or materials discussed in this manuscript.

## Appendix Supplementary materials

Image, application 1
