# Supplementary material for: Impact of physiological factors on longitudinal structural MRI measures of the brain
Source: Psychiatry Res. 2022 Apr;321:111446. doi: 10.1016/j.pscychresns.2022.111446 (PMC8924876; doi:10.1016/j.pscychresns.2022.111446)
Supplement: Supplementary file 1 [file mmc1.docx]

Supplementary Materials

Table of Contents

[S1. Scatterplots 2](#_Toc92458084)

[S2. Exploratory Regions 4](#_Toc92458085)

[S3. Maximum Percentage Change 11](#_Toc92458086)

# S1. Scatterplots

Supplementary Figure 1 Graph with the line of identity illustrating mean brain volume of the lateral ventricles for baseline and follow-up visits,

Supplementary Figure 2 Graph with the line of identity illustrating mean brain volume of the hippocampus for baseline and follow-up visits


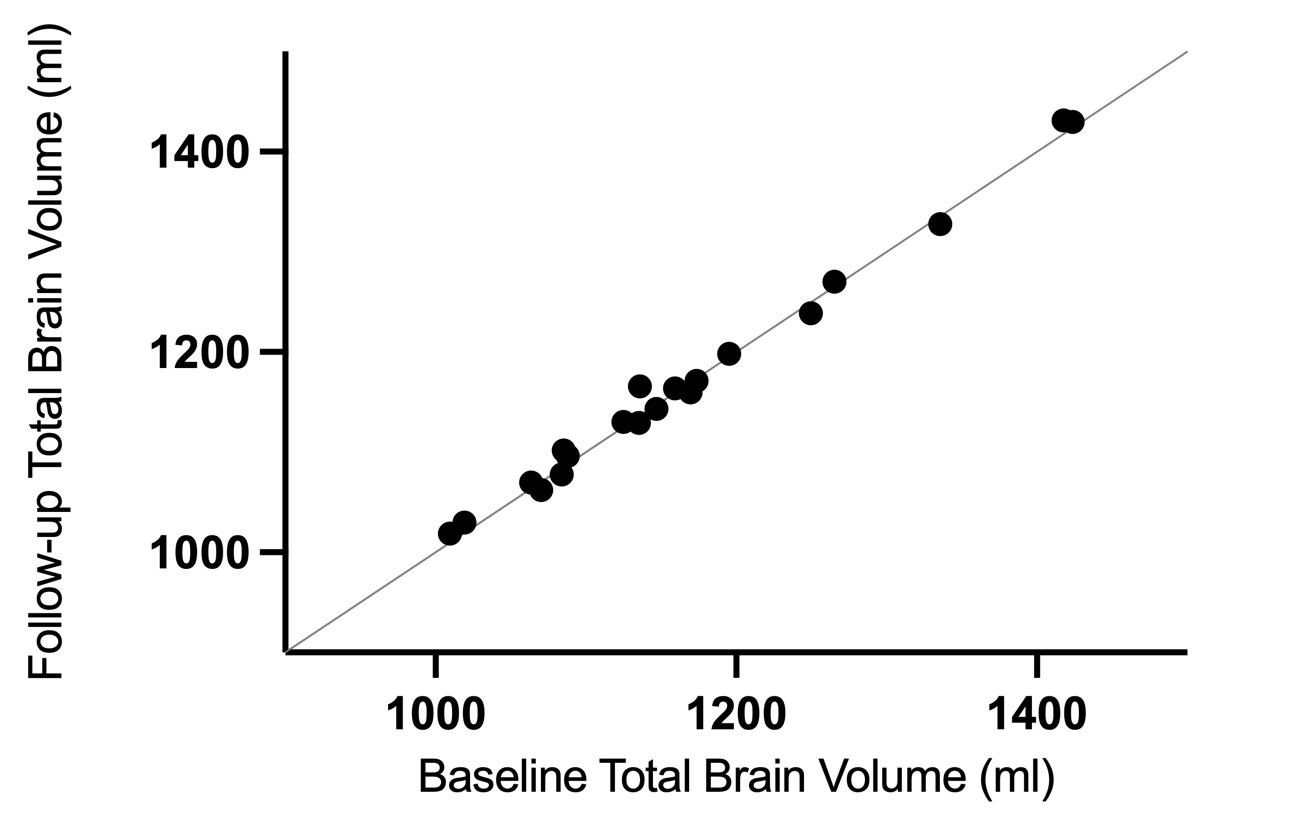


Supplementary Figure 3 Graph with the line of identity illustrating the mean brain volume of the total brain for baseline and follow-up visits

# S2. Exploratory Regions

| Thalamus (ml) | *p-value* | Coefficient^a^ | 95% Confidence interval | |
| --- | --- | --- | --- | --- |
| Hydration | 0.122 | -8.26 | -18.71 | 2.20 |
| Systolic Blood Pressure | 0.253 | 0.01 | -0.01 | 0.03 |
| Diastolic Blood Pressure | 0.543 | 0.01 | -0.01 | 0.03 |
| Caffeine | 0.305 | 0.08 | -0.07 | 0.23 |
| Time of Day | 0.518 | -0.01 | -0.06 | 0.03 |

| Caudate (ml) | *p-value* | Coefficient^a^ | 95% Confidence interval | |
| --- | --- | --- | --- | --- |
| Hydration | 0.292 | 2.67 | -2.29 | 7.63 |
| Systolic Blood Pressure | 0.886 | 0.00 | -0.01 | 0.01 |
| Diastolic Blood Pressure | 0.110 | 0.01 | 0.00 | 0.02 |
| Caffeine | 0.351 | 0.03 | -0.04 | 0.10 |
| Time of Day | **0.044*** | **0.02** | **0.00** | **0.04** |

| Putamen (ml) | *p-value* | Coefficient^a^ | 95% Confidence interval | |
| --- | --- | --- | --- | --- |
| Hydration | 0.831 | 0.92 | -7.47 | 9.30 |
| Systolic Blood Pressure | 0.788 | 0.00 | -0.02 | 0.01 |
| Diastolic Blood Pressure | 0.222 | 0.01 | -0.01 | 0.02 |
| Caffeine | 0.913 | -0.01 | -0.12 | 0.11 |
| Time of Day | 0.707 | -0.01 | -0.04 | 0.03 |

| Pallidum (ml) | *p-value* | Coefficient^a^ | 95% Confidence interval | |
| --- | --- | --- | --- | --- |
| Hydration | 0.366 | -1.65 | -5.23 | 1.93 |
| Systolic Blood Pressure | **0.017*** | **0.01** | **0.00** | **0.01** |
| Diastolic Blood Pressure | 0.200 | 0.00 | -0.00 | 0.01 |
| Caffeine | 0.917 | 0.00 | -0.05 | 0.05 |
| Time of Day | 0.060 | -0.01 | -0.03 | 0.00 |

| Amygdala (ml) | *p-value* | Coefficient^a^ | 95% Confidence interval | |
| --- | --- | --- | --- | --- |
| Hydration | 0.659 | 0.82 | -2.83 | 4.47 |
| Systolic Blood Pressure | 0.827 | 0.00 | -0.01 | 0.01 |
| Diastolic Blood Pressure | 0.579 | 0.00 | 0.00 | 0.01 |
| Caffeine | 0.474 | -0.02 | -0.07 | 0.03 |
| Time of Day | 0.573 | 0.00 | -0.01 | 0.02 |

| Accumbens Area (ml) | *p-value* | Coefficient^a^ | 95% Confidence interval | |
| --- | --- | --- | --- | --- |
| Hydration | 0.999 | 0.00 | -1.91 | 1.91 |
| Systolic Blood Pressure | 0.077 | 0.00 | 0.00 | 0.01 |
| Diastolic Blood Pressure | 0.409 | 0.00 | 0.00 | 0.00 |
| Caffeine | 0.806 | 0.00 | -0.02 | 0.03 |
| Time of Day | 0.475 | 0.00 | 0.00 | 0.01 |

| Third Ventricle (ml) | *p-value* | Coefficient^a^ | 95% Confidence interval | |
| --- | --- | --- | --- | --- |
| Hydration | **0.018*** | **1.52** | **0.26** | **2.78** |
| Systolic Blood Pressure | 0.261 | 0.00 | 0.00 | 0.00 |
| Diastolic Blood Pressure | 0.743 | 0.00 | 0.00 | 0.00 |
| Caffeine | 0.964 | 0.00 | -0.02 | 0.02 |
| Time of Day | 0.255 | 0.00 | -0.01 | 0.00 |

| Fourth Ventricle (ml) | *p-value* | Coefficient^a^ | 95% Confidence interval | |
| --- | --- | --- | --- | --- |
| Hydration | 0.197 | 1.58 | -0.82 | 3.99 |
| Systolic Blood Pressure | 0.160 | 0.00 | 0.00 | 0.01 |
| Diastolic Blood Pressure | 0.098 | 0.00 | 0.00 | 0.01 |
| Caffeine | 0.568 | 0.01 | -0.02 | 0.04 |
| Time of Day | 0.538 | 0.00 | -0.01 | 0.01 |

| Ventral  Diencephalon (ml) | *p-value* | Coefficient^a^ | 95% Confidence interval | |
| --- | --- | --- | --- | --- |
| Hydration | **0.018*** | -6.14 | **-11.24** | **-1.03** |
| Systolic Blood Pressure | 0.835 | 0.00 | -0.01 | 0.01 |
| Diastolic Blood Pressure | 0.500 | 0.00 | -0.01 | 0.01 |
| Caffeine | 0.868 | -0.01 | -0.09 | 0.07 |
| Time of Day | 0.828 | 0.00 | -0.02 | 0.03 |

| Brain Stem (ml) | *p-value* | Coefficient^a^ | 95% Confidence interval | |
| --- | --- | --- | --- | --- |
| Hydration | 0.832 | 0.80 | -6.60 | 8.21 |
| Systolic Blood Pressure | 0.961 | 0.00 | -0.01 | 0.01 |
| Diastolic Blood Pressure | 0.846 | 0.00 | -0.01 | 0.01 |
| Caffeine | 0.325 | 0.05 | -0.05 | 0.15 |
| Time of Day | 0.304 | -0.01 | -0.04 | 0.01 |

| Corpus Callosum (ml) | *p-value* | Coefficient^a^ | 95% Confidence interval | |
| --- | --- | --- | --- | --- |
| Hydration | 0.269 | -1.54 | -4.27 | 1.19 |
| Systolic Blood Pressure | 0.635 | 0.00 | 0.00 | 0.01 |
| Diastolic Blood Pressure | 0.353 | 0.00 | -0.00 | 0.01 |
| Caffeine | 0.786 | -0.01 | -0.04 | 0.03 |
| Time of Day | 0.835 | 0.00 | -0.01 | 0.01 |

| Total Brain Volume including ventricles (ml) | *p-value* | Coefficient^a^ | 95% Confidence interval | |
| --- | --- | --- | --- | --- |
| Hydration | 0.482 | 139.06 | -248.65 | 526.76 |
| Systolic Blood Pressure | 0.956 | -0.02 | -0.71 | 0.67 |
| Diastolic Blood Pressure | 0.447 | 0.26 | -0.41 | 0.94 |
| Caffeine | **0.039*** | **5.18** | **0.26** | **10.10** |
| Time of Day | 0.536 | 0.47 | -1.01 | 1.95 |

| Total Gray Matter Volume (ml) | *p-value* | Coefficient^a^ | 95% Confidence interval | |
| --- | --- | --- | --- | --- |
| Hydration | 0.366 | 147.73 | -172.48 | 467.94 |
| Systolic Blood Pressure | 0.544 | -0.17 | -0.72 | 0.39 |
| Diastolic Blood Pressure | 0.480 | 0.20 | -0.36 | 0.76 |
| Caffeine | **0.006*** | **5.36** | **1.52** | **9.20** |
| Time of Day | 0.471 | 0.45 | -0.77 | 1.68 |

| Cerebellum White Matter (ml) | *p-value* | Coefficient^a^ | 95% Confidence interval | |
| --- | --- | --- | --- | --- |
| Hydration | 0.253 | -26.42 | -71.70 | 18.86 |
| Systolic Blood Pressure | 0.385 | 0.03 | -0.04 | 0.10 |
| Diastolic Blood Pressure | 0.938 | 0.00 | -0.08 | 0.08 |
| Caffeine | 0.605 | -0.17 | -0.83 | 0.49 |
| Time of Day | 0.529 | -0.06 | -0.24 | 0.12 |

| Cerebellum Cortex (ml) | *p-value* | Coefficient^a^ | 95% Confidence interval | |
| --- | --- | --- | --- | --- |
| Hydration | 0.780 | -6.15 | \| -49.38 \| 37.09 \| \| --- \| --- \| | |
| Systolic Blood Pressure | 0.868 | 0.01 | -0.07 | 0.08 |
| Diastolic Blood Pressure | 0.238 | -0.04 | \| -0.12 \| 0.03 \| \| --- \| --- \| | |
| Caffeine | 0.551 | 0.18 | \| -0.41 \| 0.78 \| \| --- \| --- \| | |
| Time of Day | 0.948 | -0.01 | \| -0.17 \| 0.16 \| \| --- \| --- \| | |

| Cerebrospinal Fluid (ml) | *p-value* | Coefficient^a^ | 95% Confidence interval | |
| --- | --- | --- | --- | --- |
| Hydration | 0.141 | 1.61 | -0.54 | 3.76 |
| Systolic Blood Pressure | 0.200 | 0.00 | 0.00 | 0.01 |
| Diastolic Blood Pressure | **0.048*** | **0.00** | **-0.01** | **0.00** |
| Caffeine | 0.657 | 145.62 | -0.02 | 0.04 |
| Time of Day | 0.249 | 0.01 | 0.00 | 0.01 |

| Cortex Volume (ml) | *p-value* | Coefficient^a^ | 95% Confidence interval | |
| --- | --- | --- | --- | --- |
| Hydration | 0.254 | 177.29 | -127.09 | 481.68 |
| Systolic Blood Pressure | 0.583 | -0.15 | -0.67 | 0.38 |
| Diastolic Blood Pressure | 0.429 | 0.21 | -0.32 | 0.74 |
| Caffeine | **0.005*** | **5.23** | **1.57** | **8.89** |
| Time of Day | 0.373 | 0.53 | -0.64 | 1.70 |

| Cerebral White Matter Volume (ml) | *p-value* | Coefficient^a^ | 95% Confidence interval | |
| --- | --- | --- | --- | --- |
| Hydration | 0.734 | 26.45 | -126.28 | 179.17 |
| Systolic Blood Pressure | 0.157 | 0.19 | -0.07 | 0.45 |
| Diastolic Blood Pressure | 0.594 | 0.07 | -0.19 | 0.34 |
| Caffeine | 0.757 | -0.33 | -2.45 | 1.78 |
| Time of Day | 0.887 | 0.04 | -0.54 | 0.62 |

| Sub Cortical Gray Volume (ml) | *p-value* | Coefficient^a^ | 95% Confidence interval | |
| --- | --- | --- | --- | --- |
| Hydration | 0.100 | -17.22 | \| -37.77 \| 3.32 \| \| --- \| --- \| | |
| Systolic Blood Pressure | 0.675 | 0.01 | -0.03 | 0.05 |
| Diastolic Blood Pressure | 0.417 | 0.02 | -0.02 | 0.05 |
| Caffeine | 0.822 | 0.04 | -0.27 | 0.34 |
| Time of Day | 0.750 | -0.01 | -0.10 | 0.07 |

| Supratentorial Volume (without ventricles) (ml) | *p-value* | Coefficient^a^ | 95% Confidence interval | |
| --- | --- | --- | --- | --- |
| Hydration | 0.365 | 175.73 | -204.73 | 556.19 |
| Systolic Blood Pressure | 0.962 | -0.02 | -0.70 | 0.66 |
| Diastolic Blood Pressure | 0.319 | 0.34 | -0.32 | 0.99 |
| Caffeine | **0.050*** | **4.91** | **0.01** | **9.81** |
| Time of Day | 0.488 | 0.52 | -0.94 | 1.98 |

| Mean Cortical  Thickness (mm) | *p-value* | Coefficient^a^ | 95% Confidence interval | |
| --- | --- | --- | --- | --- |
| Hydration | 0.641 | 0.00 | 0.00 | 0.00 |
| Systolic Blood Pressure | 0.425 | 0.00 | 0.00 | 0.00 |
| Diastolic Blood Pressure | 0.324 | 0.00 | 0.00 | 0.00 |
| Caffeine | 0.117 | 0.00 | 0.00 | 0.00 |
| Time of Day | 0.855 | 0.00 | 0.00 | 0.00 |

| White Surface Area (mm^2^) | *p-value* | Coefficient^a^ | 95% Confidence interval | |
| --- | --- | --- | --- | --- |
| Hydration | 0.151 | 32.63 | -11.87 | 77.14 |
| Systolic Blood Pressure | 0.927 | 0.00 | -0.08 | 0.09 |
| Diastolic Blood Pressure | 0.814 | 0.01 | -0.07 | 0.09 |
| Caffeine | 0.194 | 0.41 | -0.21 | 1.03 |
| Time of Day | 0.216 | 0.11 | -0.06 | 0.28 |

# S3. Maximum Percentage Change

Although findings in the present study indicate that physiological variables did not have a substantial effect on brain volume, it is useful to estimate the maximum percentage change in brain volumes that could be expected to occur from normal variation of such physiological variables. As reported in Supplementary Table 1, the estimates for maximum percentage change in total brain volumes were computed based on the linear mixed model outputs for each physiological variable (*see* Table 3). An example of this calculation is displayed in Supplementary Figure 4, which shows that the levels of hydration may result in a total brain volume percentage change of -0.34 to 0.23%. More conservative estimates of percentage change in brain volume were also calculated using the 95% confidence intervals of the coefficients from the linear mixed model outputs, which account for the uncertainty in the gradient of the regression line.

*
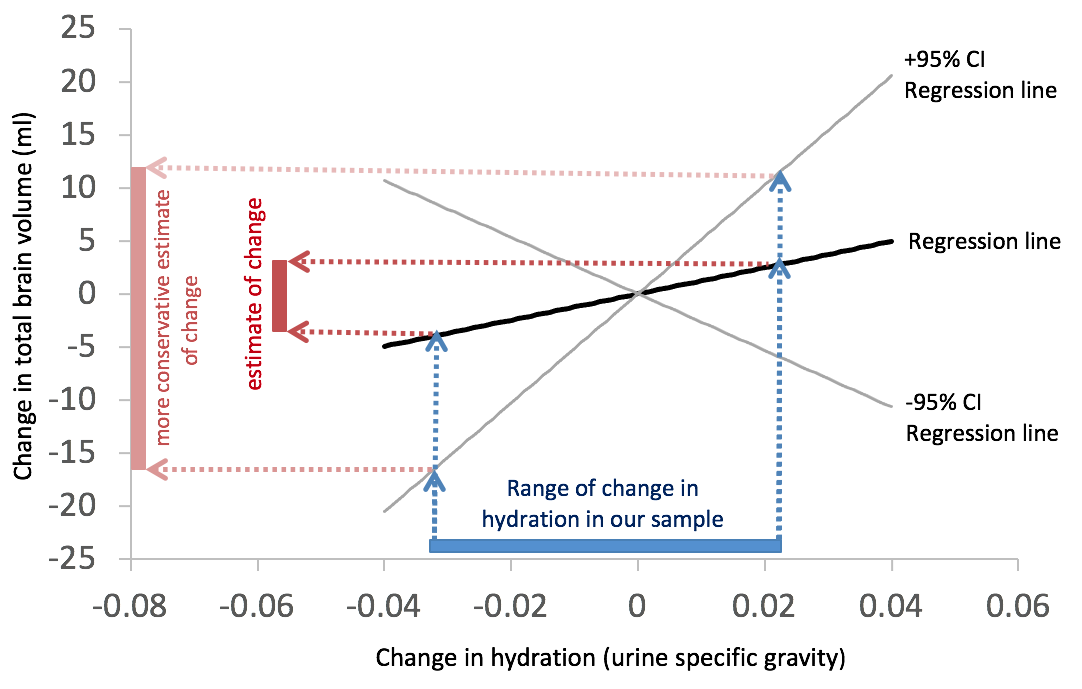
*

*Supplementary Figure 4 Graph showing the calculated regression line (black) for change in hydration and the association with change in brain volume. The gradient of the regression line is 123.57 which is the coefficient from table 3. The 95% confidence intervals are shown in grey are also from table 3. The actual range in change in hydration from baseline to follow-up in our sample is shown in blue (-0,032 to 0.022). For the estimate of brain volume change, the regression line is used to determine the change in volume (-3.9ml to 2.7ml) which considering an average total brain volume of 1168ml (table 2) is equal to (-0.34% to 0.23%). In a more conservative estimate of change, the widest 95% confidence interval is used; this corresponds to (-16.4ml to 11.3ml) which is equal to (-1.41% to 0.97%).*

|  | Hippocampus | Total Brain Volume | Lateral Ventricles |
| --- | --- | --- | --- |
| Hydration | -0.58 to 0.85 | -0.34 to 0.23 | -2.24 to 1.54 |
| Systolic Blood Pressure | -0.36 to 0.42 | -0.07 to 0.08 | -2.77 to 2.35 |
| Diastolic Blood Pressure | -0.32 to 0.52 | -0.40 to 0.24 | -0.02 to 0.01 |
| Caffeine | -0.10 to 0.20 | -0.42 to 0.84 | -1.81 to 3.63 |
| Time of Day | -1.35 to 0.64 | -0.15 to 0.31 | -2.07 to 4.36 |

*Supplementary Table 1 Estimates of largest percentage change for hypothesised brain structures from the different measures.*

|  | Hippocampus | Total Brain Volume | Lateral Ventricles |
| --- | --- | --- | --- |
| Hydration | -1.86 to 2.70 | -1.41 to 0.97 | -7.25 to 4.99 |
| Systolic Blood Pressure | -1.46 to 1.73 | -0.72 to 0.85 | -6.27 to 5.31 |
| Diastolic Blood Pressure | -1.45 to 2.38 | -1.45 to 0.89 | -5.04 to 5.00 |
| Caffeine | -1.45 to 1.84 | -0.85 to 1.70 | -3.88 to 7.76 |
| Time of Day | -3.42 to 1.62 | -0.90 to 1.53 | -4.63 to 9.77 |

Supplementary Table 2 Conservative estimate of largest percentage change for hypothesised brain structures from the different measures.
